# Inhibition of growth of OV-1063 human epithelial ovarian cancers and c- *jun* and c- *fos* oncogene expression by bombesin antagonists

**DOI:** 10.1054/bjoc.2000.1374

**Published:** 2000-09-04

**Authors:** I Chatzistamou, A V Schally, B Sun, P Armatis, K Szepeshazi

**Affiliations:** Endocrine, Polypeptide and Cancer Institute, Veterans Affairs Medical Center and Section of Experimental Medicine; Department of Medicine, Tulane University School of Medicine, New Orleans, LA 70112, USA

**Keywords:** cancer therapy, bombesin/GRP antagonists, LH-RH antagonist, ovarian tumours, c- *jun*, c- *fos*

## Abstract

Receptors for bombesin are present on human ovarian cancers and bombesin-like peptides could function as growth factors in this carcinoma. Therefore, we investigated the effects of bombesin/gastrin-releasing peptide (GRP) antagonists RC-3940-II and RC-3095 on the growth of human ovarian carcinoma cell line OV-1063, xenografted into nude mice. Treatment with RC-3940-II at doses of 10 μg and 20 μg per day s.c. decreased tumour volume by 60.9% (*P*< 0.05) and 73.5% (*P*< 0.05) respectively, after 25 days, compared to controls. RC-3095 at a dose of 20 μg per day reduced the volume of OV-1063 tumours by 47.7% (*P* = 0.15). In comparison, luteinizing hormone-releasing hormone (LH-RH) antagonist Cetrorelix at a dose of 100 μg per day caused a 64.2% inhibition (*P*< 0.05). RT-PCR analysis showed that OV-1063 tumours expressed mRNA for bombesin receptor subtypes BRS-1, BRS-2, and BRS-3. In OV-1063 cells cultured in vitro, GRP(14–27) induced the expression of mRNA for c- *jun* and c- *fos* oncogenes in a time-dependent manner. Antagonist RC-3940-II inhibited the stimulatory effect of GRP(14–27) on c- *jun* and c- *fos* in vitro. In vivo, the levels of c- *jun* and c- *fos* mRNA in OV-1063 tumours were decreased by 43% (*P*< 0.05) and 45% (*P* = 0.05) respectively, after treatment with RC-3940-II at 20 μg per day. Exposure of OV-1063, UCI-107 and ES-2 ovarian carcinoma cells to RC-3940-II at 1 μM concentration for 24 h in vitro, extended the latency period for the development of palpable tumours in nude mice. Our results indicate that antagonists of bombesin/GRP inhibit the growth of OV-1063 ovarian cancers by mechanisms that probably involve the downregulation of c- *jun* and c- *fos* proto-oncogenes. © 2000 Cancer Research Campaign


*British Journal of Cancer* (2000) **83**(7), 906–913
© 2000 Cancer Research Campaign
doi: 10.1054/ bjoc.2000.1374, available online at http://www.idealibrary.com on IDEAL®

### Inhibition of growth of OV-1063 human epithelial ovarian cancers and c-*jun* and c-*fos* oncogene expression by bombesin antagonists

**I Chatzistamou[1,2], AV Schally[1,2], B Sun[1,2], P Armatis[1] and K Szepeshazi[1,2]**

[1] Endocrine, Polypeptide and Cancer Institute, Veterans Affairs Medical Center, and Section of Experimental Medicine; [2]Department of Medicine, Tulane University School of Medicine, New Orleans, LA 70112, USA

**Summary** Receptors for bombesin are present on human ovarian cancers and bombesin-like peptides could function as growth factors in this carcinoma. Therefore, we investigated the effects of bombesin/gastrin-releasing peptide (GRP) antagonists RC-3940-II and RC-3095 on the growth of human ovarian carcinoma cell line OV-1063, xenografted into nude mice. Treatment with RC-3940-II at doses of 10 μg and 20 μg per day s.c. decreased tumour volume by 60.9% ($P < 0.05$) and 73.5% ($P < 0.05$) respectively, after 25 days, compared to controls. RC-3095 at a dose of 20 μg per day reduced the volume of OV-1063 tumours by 47.7% ($P = 0.15$). In comparison, luteinizing hormone-releasing hormone (LH-RH) antagonist Cetrorelix at a dose of 100 μg per day caused a 64.2% inhibition ($P < 0.05$). RT-PCR analysis showed that OV-1063 tumours expressed mRNA for bombesin receptor subtypes BRS-1, BRS-2, and BRS-3. In OV-1063 cells cultured in vitro, GRP(14–27) induced the expression of mRNA for c-*jun* and c-*fos* oncogenes in a time-dependent manner. Antagonist RC-3940-II inhibited the stimulatory effect of GRP(14–27) on c-*jun* and c-*fos* in vitro. In vivo, the levels of c-*jun* and c-*fos* mRNA in OV-1063 tumours were decreased by 43% ($P < 0.05$) and 45% ($P = 0.05$) respectively, after treatment with RC-3940-II at 20 μg per day. Exposure of OV-1063, UCI-107 and ES-2 ovarian carcinoma cells to RC-3940-II at 1 μM concentration for 24 h in vitro, extended the latency period for the development of palpable tumours in nude mice. Our results indicate that antagonists of bombesin/GRP inhibit the growth of OV-1063 ovarian cancers by mechanisms that probably involve the downregulation of c-*jun* and c-*fos* proto-oncogenes. © 2000 Cancer Research Campaign

**Keywords:** cancer therapy; bombesin/GRP antagonists; LH-RH antagonist; ovarian tumours; c-*jun*; c-*fos*

Ovarian epithelial carcinoma is the leading cause of death from gynaecological cancers among women in the western world. It is estimated that in 1999 approximately 25 000 women in the USA will have been diagnosed with ovarian cancer and about 14 000 deaths will have occurred due to this malignancy (Landis et al, 1999). Over the period 1990–1994 nearly 20 000 deaths from ovarian cancer were recorded in the UK (Levi et al, 1999). Most patients with advanced epithelial ovarian cancer are presently treated with cytoreductive surgery followed by combination chemotherapy. However, the long-term outcome of such treatment is disappointing and new therapeutic strategies must be explored.

Bombesin-like peptides, such as the gastrin-releasing peptide (GRP), were initially reported as autocrine growth factors in the development and progression of some human small cell lung carcinomas (Cuttitta et al, 1985; Carney et al, 1987; Moody and Cuttitta, 1993; Siegfried et al, 1994), but recent studies also suggest an involvement of bombesin and GRP in the pathogenesis of pancreatic, prostatic, breast and other cancers, such as malignant glioblastomas (Bologna et al, 1989; Yano et al, 1992; Wang et al, 1996; Schally and Comaru-Schally, 1997; Kiaris et al, 1999; Markwalder and Reubi, 1999). Specific receptors for bombesin/GRP have been shown in various human cancers, including prostatic and mammary, in human breast, prostatic and pancreatic cancer lines, and in mouse mammary cancers (Szepeshazi et al, 1992; 1997; Halmos et al, 1995; Wang et al, 1996; Schally and Comaru-Schally, 1997; Markwalder and Reubi, 1999; Sun et al, 1999). Recently, the expression of receptors for bombesin-like peptides was demonstrated in human ovarian cancer specimens and ovarian cancer cell lines SW-626, OV-1063 and UCI-107 (Schally and Comaru-Schally, 1997; Kim et al, 1998; Sun et al, 1999). Until now, three subtypes of bombesin/GRP receptors have been characterized in humans: bombesin receptor subtype-1 (GRPR/BRS-1) which binds GRP with high affinity; NMBR/BRS-2, which is preferentially activated by neuromedin B; and BRS-3 (Spindel et al, 1993). BRS-3 is considered an orphan receptor with an unknown natural ligand. Recently, a fourth receptor subtype BRS-4 has been cloned and characterized in amphibia and its existence in mammals was also postulated (Nagalla et al, 1995). The peptides of bombesin/GRP family induce cell proliferation by mechanisms that involve activation of phosphatidylinositol, $Ca^{2+}$ release, and stimulation of the expression of c-*fos* and c-*jun* mRNAs (Spindel et al, 1993; Draoui et al, 1995; Nagalla et al, 1995).

The findings that bombesin-like and gastrin-like peptides may function as autocrine/paracrine growth factors in certain tumours prompted the development of bombesin/GRP antagonists as potent antitumour agents (Radulovic et al, 1991; Cai et al, 1994; Reile et al, 1995; Schally and Comaru-Schally, 1997). Bombesin/GRP antagonists, such as RC-3095 and RC-3940-II, synthesized in our laboratory suppressed the growth of various tumours including prostatic, breast, lung, pancreatic, gastric and malignant glioblastomas (Szepeshazi et al, 1992; 1997; Qin et al, 1994*a*; 1994*b*;

*Received 29 November 1999*
*Revised 17 May 2000*
*Accepted 23 May 2000*

*Correspondence to:* AV Schally

Shirahige et al, 1994; Jungwirth et al, 1997*a*; 1997*b*; Koppan et al, 1998; Miyazaki et al, 1998; Kiaris et al, 1999). RC-3095 and RC-3940-II differ at N-terminus and at C-terminal. RC-3095 contains N-terminal D-Tpi and C-terminal Leu, and RC-3940-II has Hca at N-terminus and Tac at C-terminus (Hca is desaminophenylalanine; Tac is thiazolidine-4-carboxylic acid; Tpi is 2,3,4,9-tetrahydro-1H-pyrido[3,4-b]indol-3-carboxylic acid). Due to their structural differences, RC-3940-II has a more restricted conformation than RC-3095 resulting in about 200 times higher binding affinity to GRP receptor than RC-3095, as demonstrated by the displacement of [$^{125}$I-Tyr$^{4}$]bombesin in Swiss 3T3 cells (Reile et al, 1995).

The receptors for luteinizing hormone-releasing hormone (LH-RH) are also expressed by ovarian cancers (Yano et al, 1994; Chegini et al, 1996; Emons et al, 1998). Our group has previously demonstrated that chronic treatment with the LH-RH antagonist Cetrorelix, but not LH-RH agonist triptorelin, can induce a significant inhibition of growth of OV-1063 human epithelial ovarian cancer xenografted into nude mice (Yano et al, 1994). Because both LH-RH analogues cause a comparable suppression of the pituitary-gonadal axis, it was suggested that the antitumour action of Cetrorelix was exerted in part directly on LH-RH receptors in tumours (Yano et al, 1994).

In the present study we evaluated the anti-tumour effects of two bombesin/GRP antagonists, RC-3095 and RC-3940-II on the growth of human epithelial ovarian cancer cell line OV-1063, xenografted into nude mice. The results were compared to those obtained with the LH-RH antagonist Cetrorelix. In an attempt to elucidate the mechanism of action of bombesin/GRP antagonists, we investigated the effect of GRP on the expression of c-*jun* and c-*fos* mRNAs in OV-1063 cells cultured in vitro. The outcome of treatment with RC-3940-II on the mRNA levels of c-*jun* and c-*fos* was also evaluated in OV-1063 tumours xenografted into nude mice.

### MATERIALS AND METHODS

### Peptides

Bombesin antagonist D-Tpi$^{6}$, Leu$^{13}\psi$[CH$_2$NH]Leu$^{14}$BN(6–14) (RC-3095), originally synthesized in our laboratory by solid-phase methods (Radulovic et al, 1991), was manufactured by ASTA Medica (Frankfurt am Main, Germany) in the form of acetate salt, D22213. The novel BN antagonist Hca$^{6}$,Leu$^{13}\psi$[CH$_2$N]Tac$^{14}$-BN (6–14) (RC-3940-II) was synthesized by solid-phase methods and purified in our laboratory (Cai et al, 1994; Reile et al, 1995). Hca is desaminophenylalanine, Tac is thiazolidine-4-carboxylic acid, and Tpi is 2,3,4,9-tetrahydro-LH-pyrido[3,4-b]indol-3-carboxylic acid. Cetrorelix (SB-75), [Ac-D-Nal(2)$^{1}$, D-Phe(4Cl)$^{2}$, D-Pal(3)$^{3}$, D-Cit$^{6}$,D- Ala$^{10}$] LHRH, originally synthesized in this laboratory (Bajusz et al, 1988), was obtained from ASTA Medica.

RC-3095 and RC-3940-II were dissolved in dimethyl sulphoxide (DMSO) and diluted with 0.9% saline. The final concentration of DMSO was 0.1%. Cetrorelix was dissolved in distilled water containing 5% mannitol.

### Cell lines and cell proliferation assays

Human ovarian cancer cell line OV-1063, and ES-2 were obtained from American Type Culture Collection (Rockville, MD, USA). UCI-107 human ovarian carcinoma cell line was kindly provided by Dr A Manetta (University of California, Irvine, CA, USA). OV-1063 and UCI-107 cells were maintained in Roswell Park Memorial Institute 1640 (RPMI-1640) medium, and ES-2 cells in McCoy 5A medium, all supplemented with 10% foetal bovine serum (FBS), vitamins, antibiotics, and antimycotics as described previously (Horowitz et al, 1985). Cells were cultured in T-75 Flasks (Corning Costar Corp., Cambridge, MA, USA) in a humidified atmosphere of 5% $CO_2$ and 95% air at 37°C and passed every 4–6 days using 0.25% trypsin-EDTA (Yano et al, 1994). For the evaluation of the c-*jun* and c-*fos* mRNA levels, cells were cultured for 18 h as described previously, except that the tissue culture medium contained 2% FBS during the exposure to GRP or RC-3940-II at $10^{-7}$ M for 1–5 h. The effect of RC-3940-II on the rate of cell proliferation was evaluated by the crystal violet method as described previously (Bernhardt et al, 1992). The results were calculated as %T/C, where T = optical density of treated cultures and C = optical density of untreated cultures.

### Studies on tumorigenicity

OV-1063, ES-2 and UCI-107 cells were exposed in vitro to $10^{-6}$ M RC-3940-II for 24 h. Subsequently, $2 \times 10^{5}$ cells per animal were injected s.c. into the right flanks of nude mice and the period during which palpable tumours, measuring about 15 mm$^{3}$, developed was recorded. Animals were observed daily until tumours were developed in all experimental animals.

### Histological methods

Histological analyses were performed in OV-1063 tumours xenografted into nude mice. Mitotic and apoptotic cells were counted in tumour slides stained with haematoxylin-eosin as described earlier (Szepeshazi et al, 1992) and their number per 1000 cells were accepted as the mitotic and apoptotic indices. For the demonstration of the nucleolar organizer regions (NOR) in tumour cell nuclei, the AgNOR method was used as described previously (Szepeshazi et al, 1992).

Proliferating cell nuclear antigen (PCNA) was detected by immunohistochemistry as follows. Sections from paraffin-embedded tumour samples were placed on silanated slides, dewaxed and rehydrated. Slides were immersed in distilled water and heated in a domestic microwave oven for $2 \times 5$ min. All incubations were performed at room temperature as follows: blocking solution 3% bovine serum albumin for 30 min, monoclonal anti-PCNA (Calbiochem, La Jolla, CA, USA), at 1:500 for 60 min, biotinylated anti-mouse IgG (Sigma, St. Louis, MO, USA), 1:400 for 60 min and Extravidine-peroxidase (Sigma), 1:100 for 60 min. The product was visualized by 3,3′-diaminobenzidine (Sigma Fast DAB). Sections were evaluated at areas showing the highest positivity. The positive and negative nuclei were counted in three microscopic areas each containing about 330 cells, and the percentage ratio of positive cells to total cells was calculated.

### Animals

Five to 6-week-old female athymic Ncr nu/nu nude mice were obtained from Frederick Cancer Research facility of the National Cancer Institute (Frederick, MD, USA). The mice were housed in sterile cages under laminar flow hoods in a temperature-controlled

© 2000 Cancer Research Campaign room with a 12-h dark schedule and were fed autoclaved chow and water ad libitum. Their care was in accord with institutional ethical guidelines for the welfare of animals.

### Experimental protocol

Xenografts of OV-1063 cells were initiated by s.c. injection of $10^7$ cells into the right flank of three nude mice. Tumours resulting after 4 weeks were aseptically dissected and mechanically minced: 1 $mm^3$ tumour pieces were transplanted subcutaneously by trocar needle into the right flank of the mice. The tumour take rate was nearly 90%. Two weeks after tumour transplantation and while the tumours measured approximately 30–40 $mm^3$, the mice were divided into five experimental groups of seven animals each and received the following treatment as s.c. injections: group 1 (control), vehicle solution; group 2, RC-3095 at a dose of 20 µg per day; group 3, RC-3940-II at a dose of 20 µg per day; group 4, RC-3940-II at a dose of 10 µg per day; group 5, Cetrorelix (SB-75) at a dose of 100 µg per day. The treatment was continued for 25 days. The dose of the analogues was determined on the basis of previous studies using different tumour models. Tumours were measured once a week with a microcaliper and the volume was calculated by the formula of length × width × height × 0.5236 (Tomayko and Reynolds, 1989). At the end of the experiment, mice were anaestaetized with methoxyflurane (Metofane; Pitman-Moore, Mundelein, IL, USA) and sacrificed by decapitation. Trunk blood was collected and centrifuged at 1000 ***g*** for 30 min at 4°C and serum was stored at –20°C until assayed. Body weights were recorded and various organs were removed and weighed. Tumours were carefully removed, cleaned and weighed. Tumour burden at the end of the experiment was calculated as tumour weight (mg) per body weight (g). Tumour pieces were stored at –80°C for molecular biology analysis. All experiments were approved by the institutional ACUC and the procedures were essentially in accordance with UKCCCR guidelines (1998) for the welfare of animals in experimental neoplasia.

### RNA extraction

Total RNA was extracted from frozen tissue samples by using RNAsol B (TEL-TEST Inc., Friendswood, TX, USA) according to the manufacturer's instructions. The RNA pellets were suspended in 50 µl of 10 mM Tris, 1 mM EDTA buffer (pH 8.0) and quantified spectrophotometrically at 260 nm.

### Reverse transcription-PCR (RT-PCR)

One microgram of total RNA was used in a test tube containing 10 mM Tris-HCI (pH 8.3), 50 mM KCI, 5 mM $MgCI_2$, 1 mM of each deoxynucleoside triphosphate, 1 U Rnase inhibitor, and 2.5 µM random hexamer primers in a final volume of 19 µl of Rnase-free deionized distilled water. The mixture was heated for 10 min at 65°C, quenched on ice, then 2.5 U of Moloney murine leukaemia virus reverse transcriptase (Perkin-Elmer Corp., Norwalk, CT, USA) in 1 µl was added, for a total reaction volume of 20 µl. The mixture was incubated at room temperature for 10 min and then at 42°C for 1 h. The reaction was ended by heating at 95°C for 5 min and quenching on ice. The PCR amplification of the cDNAs for human glyceraldehyde-3-phosphate dehydrogenase (hGAPDH), GRP, c-*fos*, c-*jun*, GRPR, BRS-3 and NMBR, was performed as follows. One microlitre of the cDNA was amplified in a 50 µl solution containing 10 mM Tris.HCl (pH 8.3), 50 mM KCl, 1.7 mM $MgCl_2$, 200 µM of each dNTP, 2.5 Units Taq polymerase and 0.4 µM of each primer. The nucleotide sequence and the expected PCR products for the oligonucleotide primers used are shown in Table 1 (Kiaris et al, 1999; Sun and Schally, 1999). PCR consisted of one cycle at 95°C for 3 min, 58°C for 1 min, and 72°C for 1 min and subsequently 26 (hGAPDH), 30 (GRP), 29 (c-*jun*), 32 (c-*fos*), cycles of 95°C for 35 s, 58°C for 40 s, and 72°C for 40 s by using a Stratagene Robocycler 40 System. For the multiplex PCRs, the cDNA for each target gene was amplified simultaneously with the cDNA for hGAPDH, after supplementation of the primers for hGAPDH at the appropriate cycle at 95°C. For the detection of GRP, after the first round of PCR, 1 µl of the PCR product was subjected to a second round of PCR consisting of 28 cycles. All other parameters for the second round of PCR amplification were similar to those described above for the first round of PCR amplification. PCR amplification for GRPR, NMBR, and BRS-3 was performed in Perkin Elmer DNA thermal cycler model 2400. Samples were denatured at 94°C for 5 min and then subjected to 40 cycles comprised of 94°C for 1 min, 52°C for 1 min and 72°C for 1 min for GRPR and BRS-3 (1 µl products of the first PCR amplification were subjected to additional 30 cycles by using nested primers); 40 cycles of 94°C for 30 s, 62°C for 30 s and 72°C for 30 s for NMBR, 25 cycles of 94°C for 30 s, 60°C for 30 s and 72°C for 30 s for hGAPDH, followed by a final extension at 72°C for 5 min. In all RT-PCR amplifications negative controls were included, in which $H_2O$ instead of RNA was used as template. Aliquots of PCR-amplified product were resolved by electrophoresis on a 1.8%

**Table 1** Nucleotide sequence and size of the expected PCR products for oligonucleotide primers used for RT-PCR

| Gene | Sequence | PCR product (bp) |
|---|---|---|
| hGAPDH | 5′-TCCTCTGACTTCAACAGCGACACC-3′ | 207 |
| | 5′-TCTCTCTTCCTCTTGTGCTCTTGG-3′ | |
| GRP | 5′-TGCAAGAATTTGCTGGGTCTC-3′ | 485 |
| | 5′-TGTGAATGGTAACAGCTGGGG-3′ | |
| c-*fos* | 5′-AAGGAGAATCCGAAGGGAAAGGAATAAGATGGCT-3′ | 612 |
| | 5′-AGACGAAGGAAGACGTGTAAGCAGTGCAGCT-3′ | |
| c-*jun* | 5′-GCATGAGGAACCGCATCGCTGCCTCCAAGT-3′ | 409 |
| | 5′-GCGACCAAGTCCTTCCCACTCGTGCACACT-3′ | |
| GRPR | 5′-ATTTGGCAGGATTGGCTGC-3′ | 158 |
| | 5′-TGAGGCAGATCTTCATCAG-3′ | |
| BRS-3 | 5′-GCTCTGTGGTTTCTAACG-3′ | 375 |
| | 5′-CTGCCTTGTATCTGTCAGC-3′ | |
| NMBR | 5′-CGGACTTCTGCTGGAAAGGA-3′ | 484 |
| | 5′-GACGTCTGCATGTCCATGG-3′ | |

 © 2000 Cancer Research Campaign agarose gel, stained with ethidium bromide, and visualized under ultraviolet light. For quantitation of PCR-amplified products, a scanning densitometer (model GS-700, Bio-Rad) was used, coupled with the Bio-Rad personal analysis software.

### Statistical analysis

Data are expressed as mean ± SE. Statistical analyses were performed using Duncan's new multiple range test (Steel and Torrie, 1976) and Student's two-tailed *t*-test, one-way Anova and Dunnett's test. All *P*-values are based on two-sided hypothesis testing.

### RESULTS

### Effects of treatment with bombesin/GRP antagonists RC-3095 and RC-3940-II and LH-RH antagonist Cetrorelix on OV-1063 tumours in nude mice

After 25 days of treatment, the volume of OV-1063 tumours in the two groups receiving the bombesin/GRP antagonist RC-3940-II, was significantly reduced to 749.1 ± 141 $mm^3$ ($P < 0.05$) for the high-dose group (20 µg per day) and to 1102.5 ± 151 $mm^3$ ($P < 0.05$) for the low-dose group (10 µg per day) as compared with the controls (2820.4 ± 348.6 $mm^3$), corresponding to decreases of 73.5% and 60.9% in tumour volume, respectively (Table 2, Figure 1). OV-1063 tumours in mice treated with the bombesin/GRP antagonist RC-3095 measured 1481 ± 531.4 $mm^3$, indicating a 47.7% decrease in tumour volume ($P = 0.15$). In the group treated with LH-RH antagonist Cetrorelix, the tumour volume was reduced to 1009.3 ± 249.2 $mm^3$, corresponding to a 64.2% ($P < 0.05$) decrease. The final tumour weights were also significantly diminished by 58.8% and 45.3% in the groups treated with the high-dose ($P < 0.05$) and the low-dose of RC-3940-II ($P < 0.05$), respectively. The tumour weight reduction in the group injected with Cetrorelix was 52.4% ($P < 0.05$) and in the group receiving RC-3095, 30.9% (not significant), as compared to controls (Table 2). A significant ($P < 0.01$) decrease in the weight of ovaries was observed in the group that received Cetrorelix. No significant differences in body weights and the weights of various organs such as liver, kidneys, and heart were observed between the control and the treated animals.

### Effect of bombesin/GRP antagonists RC-3095 and RC-3940-II on the rate of cell proliferation

Cells cultured in vitro were exposed to bombesin/GRP antagonists at various concentrations and their proliferation was monitored. Using the crystal violet method, no significant effect was found on the proliferation rate of OV-1063, ES-2 and UCI-107 ovarian carcinoma cells after exposure to RC-3095 or RC-3940-II at $10^{-7}$–$10^{-5}$M (data not shown).

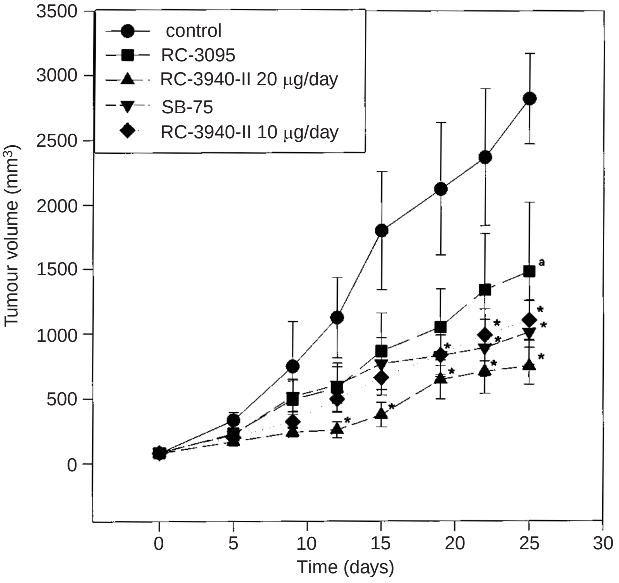

**Figure 1** Tumour volumes in athymic female nude mice bearing s.c. transplanted OV-1063 human epithelial ovarian cancer cell line during treatment with bombesin/GRP antagonists RC-3940-II at doses of 10 and 20 µg per animal and RC-3095 at a dose of 20 µg per animal, and LH-RH antagonist Cetrorelix at a dose of 100 µg per animal administered by daily s.c. injections. Vertical bars represent SE; *$P < 0.05$ vs control; $^aP = 0.15$

### Effect of bombesin/GRP antagonist RC-3940-II on tumorigenicity of ovarian cancer cells

Before the injection into nude mice, OV-1063, UCI-107 and ES-2 ovarian carcinoma cells were exposed to RC-3940-II for 24 h at $10^{-6}$ M and the number of animals bearing palpable tumours was recorded daily. As shown in Figure 2, the latency period for the development of palpable tumours was extended by the pre-treatment with RC-3940-II from 11.1 ± 1.0 days to 14.6 ± 1.0 days for OV-1063 cells, from 7.5 ± 0.9 days to 13 ± 0.8 days for UCI-107 cells and from 7.9 ± 1.5 days to 14 ± 1.0 days for ES-2 cells. These findings indicated that the latency period for the establishment of OV-1063, UCI-107 and ES-2 xenografts into nude mice was significantly extended by 32% ($P < 0.05$ vs controls), 73% ($P < 0.001$ vs controls) and 77% ($P < 0.01$ vs controls), respectively.

**Table 2** Effect of treatment with bombesin/GRP antagonists RC-3095 and RC-3940-II and LH-RH antagonist Cetrorelix on tumour volume and weight in nude mice bearing xenografts of OV-1063 human epithelial ovarian cancer cell line

| Treatment | Intitial tumour volume ($mm^3$) | Final tumour volume ($mm^3$) | Tumour weight (g) | Tumour burden (mg $g^{-1}$ bw) |
|---|---|---|---|---|
| Control | 79.3 ± 17.4 | 2820.4 ± 348.6 | 4.88 ± 0.67 | 146.0 ± 19.0 |
| RC-3095 | 81.0 ± 23.4 | 1481.4 ± 531.4 | 3.37 ± 0.87 | 107.0 ± 25.9 |
| RC-3940-II/ 20 µg $ml^{-1}$ | 78.3 ± 12.8 | 749.1 ± 141.0[a] | 2.01 ± 0.35[a] | 72.4 ± 13.3[a] |
| RC-3940-II/ 10 µg $ml^{-1}$ | 81.3 ± 11.2 | 1102.5 ± 151.0[a] | 2.67 ± 0.41[a] | 89.9 ± 13.7[a] |
| Cetrorelix | 86.5 ± 14.5 | 1009.3 ± 249.2[a] | 2.32 ± 0.61[a] | 76.6 ± 16.7[a] |

[a] $P < 0.05$

© 2000 Cancer Research Campaign

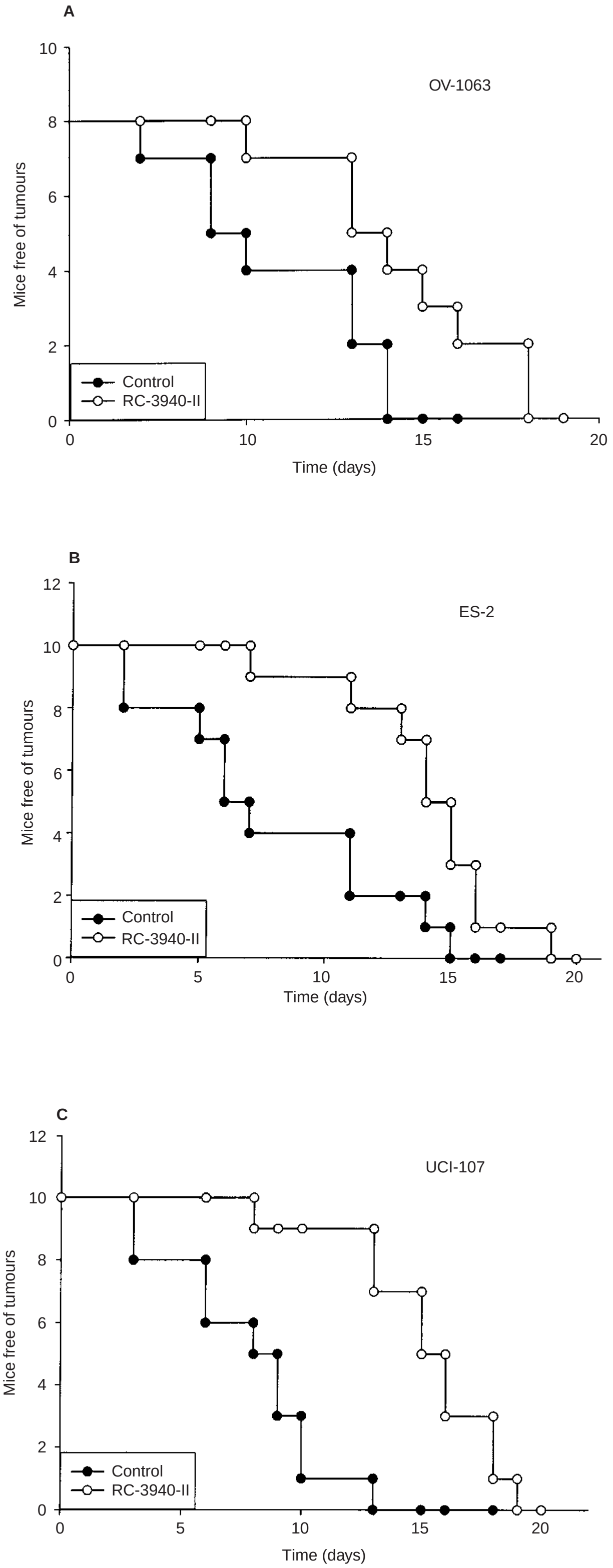

**Figure 2** Effect of bombesin/GRP antagonist RC3940-II on the tumorigenicity of OV-1063 (**A**), ES-2 (**B**) and UCI-107 (**C**) ovarian carcinoma cells. Cells were exposed in vitro to RC-3940-II at $10^{-6}$ M for 24 h and subsequently injected s.c. into nude mice at $2 \times 10^5$ cells per animal. The number of the tumour-bearing animals was recorded daily until all animals developed palpable tumours

### Histological findings

The results of the histological analysis are summarized in Table 3. Bombesin/GRP antagonist RC-3940-II administered at 10 µg per day or at 20 µg per day decreased significantly ($P < 0.05$) the AgNOR numbers in the OV-1063 tumours as compared with the controls, while RC-3095 administered at 20 µg per day did not cause any significant reduction. PCNA expression was decreased only by Cetrorelix and RC-3940-II (at doses of 20 µg per day) ($P < 0.05$). Cetrorelix was also the only antagonistic analogue that increased significantly ($P < 0.05$) the apoptotic index and the ratio of apoptotic to mitotic indices in OV-1063 tumours, compared to controls. PCNA indices showed significant correlation with AgNOR counts ($r = 0.556$, $P = 0.013$), but not with mitotic indices.

### Investigation of the expression of mRNA for GRP and bombesin receptor subtypes in OV-1063 human ovarian epithelial cell carcinoma

The expression of mRNA for GRP and bombesin receptor subtypes BRS-1 (GRPR), BRS-2 (NMBR) and BRS-3 in OV-1063 tumours was evaluated by RT-PCR. As shown in Figure 3, mRNA for GRPR, NMBR and BRS-3 was detected in OV-1063 tumours, but no mRNA for the GRP ligand could be found. To confirm the absence of expression of mRNA for GRP, PCR products were subjected to a second round of PCR amplification which was again negative for the expected 485 bp band (Figure 4).

### Effect of GRP and bombesin/GRP antagonist RC-3940-II on mRNA expression of c-*jun* and c-*fos* oncogenes in vitro and in vivo

In an attempt to investigate further the mechanism of anti-tumour action of bombesin/GRP antagonists, we studied the role of GRP(14–27) on the expression of mRNA for c-*jun* and c-*fos* oncogenes, in vitro. The mRNA levels of c-*jun* and c-*fos* oncogenes were assessed by RT-PCR after exposure of OV-1063 cells cultured in vitro to $10^{-7}$ M GRP(14–27) for 1 h, 3 h and 5 h. As shown in Figure 5, the maximal stimulation of c-*jun* mRNA levels, about 436% vs basal, was observed 1 h after the exposure to GRP(14–27), while the greatest increase in c-*fos* mRNA levels, about 169% vs basal, was obtained after 5 h incubation with GRP(14–27). The stimulation of mRNA for c-*jun* and c-*fos* by GRP(14–27) was suppressed in the presence of $10^{-7}$ M bombesin/GRP antagonist RC-3940-II. In vivo, the treatment of mice bearing OV-1063 xenografts with RC-3940-II at a dose of 20 µg per day resulted in a significant decrease of 43% ($P < 0.05$) and 45% ($P < 0.05$) in the mRNA levels for c-*jun* and c-*fos* oncogenes respectively, while RC-3040-II at 10 µg per day had no effect (Figure 6).

### DISCUSSION

The present study shows for the first time that an antagonist of bombesin/GRP, RC-3940-II, can significantly inhibit the growth of OV-1063 human ovarian epithelial cancers xenografted into nude mice when administered at doses of 10 µg and 20 µg per day. The anti-tumour action of RC-3940-II is in agreement with the decrease in the expression of PCNA and AgNOR numbers in OV-1063 tumours xenografted into nude mice. PCNA and AgNOR are

 © 2000 Cancer Research Campaign

**Table 3** Effect of treatment with bombesin/GRP antagonists RC-3095 and RC-3940-II and LH–RH antagonist Cetrorelix on various histological parameters of OV-1063 human ovarian carcinomas xenografted into nude mice

| Groups | Mitotic index | Apoptotic index | Ratio apoptotic: mitotic indices | AgNORs per cell (*n*) | PCNA index (%) |
|---|---|---|---|---|---|
| Control | 14.0 ± 2.9 | 3.4 ± 0.4 | 0.29 ± 0.08 | 6.65 ± 0.17 | 89.1 ± 0.8 |
| RC-3940-II/ 20 µg | 12.3 ± 1.3 | 4.5 ± 0.9 | 0.40 ± 0.13 | 5.85 ± 0.19[a] | 83.1 ± 0.7[a] |
| RC-3940-II/ 10 µg | 7.9 ± 0.7 | 5.6 ± 0.7 | 0.72 ± 0.06 | 5.93 ± 0.18[a] | 85.3 ± 1.4 |
| RC-3095 | 14.5 ± 0.9 | 4.3 ± 0.7 | 0.30 ± 0.06 | 6.00 ± 0.06 | 86.7 ± 1.0 |
| SB-75 | 12.5 ± 1.7 | 10.4 ± 1.3[a] | 0.90 ± 0.21[a] | 5.95 ± 0.13[a] | 84.8 ± 0.7[a] |

Values are means ± SE. [a]$P < 0.05$ vs control

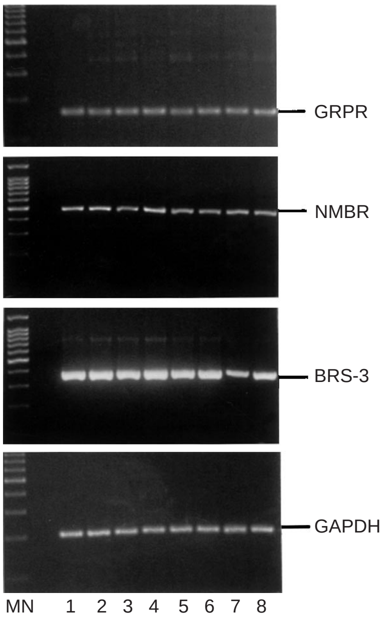

**Figure 3** Expression of bombesin receptor subtypes in OV-1063 human ovarian cancers. Lanes 1, 2 = control groups; lanes 3,4 = groups treated with RC-3940-II (20 µg per day); lanes 5, 6 = groups treated with RC-3940-II (10 µg per day); lane 7, 8 = groups treated with RC-3095; M = DNA molecular marker; N = negative control

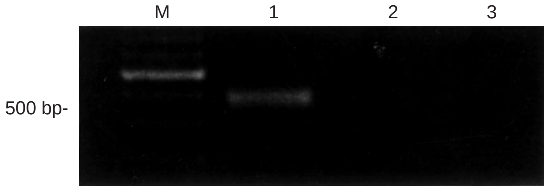

**Figure 4** Expression of mRNA for GRP ligand. Lane 1 = positive control (RNA isolated from NCI-H-69 small cell lung carcinoma); lane 2 = RNA isolated from OV-1063 human ovarian epithelial cancer cell line; lane 3 = negative control

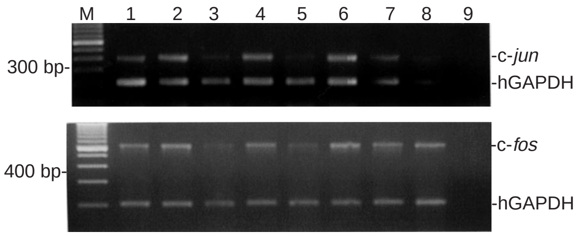

**Figure 5** Expression of c-*jun* and c-*fos* mRNA in OV-1063 human ovarian epithelial cancer cell line. Total RNA was extracted from cells cultured in vitro, and c-*jun* and hGAPDH as well as c-*fos* and hGAPDH were co-amplified by multiplex RT-PCR and electrophoresed on 2% agarose. Each RT-PCR reaction was repeated at least three times and similar results were obtained. Lane 1 = expression of c-*jun* and c-*fos* mRNA in cells without pre-treatment; lanes 2, 4, 6 = expression of c-*jun* and c-*fos* in cells treated with $10^{-7}$ MGRP(14–27) for 1 h, 3 h, and 5 h; lanes 3, 5, 7 = expression of c-*jun* and c-*fos* mRNA in cells treated with a mixture of $10^{-7}$ M RC-3940-II and $10^{-7}$ M GRP(14–27); lane 8 = expression of c-*jun* and c-*fos* in cells treated with RC-3940-II alone; lane 9 = negative control; M = DNA molecular size marker markers of cell proliferation and their expression is increased in highly proliferative tissues. RC-3940-II also extended significantly the latency period for the development of palpable tumours in OV-1063, ES-2 and UCI-107 human ovarian carcinoma cells. This finding was most likely due to the direct effect on tumorigenicity and not to the cytotoxicity of bombesin/GRP antagonists on these cells, because exposure of OV-1063, ES-2 and UCI-107 cells cultured in vitro to RC-3940-II had no effect on the rate of cell proliferation. Another antagonist of bombesin/GRP, RC-3095, previously developed in our laboratory, was less potent than RC-3940-II in inhibiting the growth of OV-1063 tumours. The anti-tumour effects of bombesin/GRP antagonists RC-3095 and RC-3940-II were also compared with the effects of LH-RH antagonist Cetrorelix, which has been previously shown to inhibit the proliferation of this tumour (Yano et al, 1994). Our results showed that RC-3940-II was marginally, but not significantly, more potent than Cetrorelix in inhibiting OV-1063 tumour growth. Treatment with Cetrorelix significantly enhanced apoptosis and increased the ratio of apoptotic to mitotic indices, which is in agreement with previous findings on the regulation of apoptosis by LH-RH analogues. That only Cetrorelix, and not RC-3940-II, induced apoptosis in OV-1063 tumours, while both significantly inhibited tumour growth to similar levels, is probably due to differences in the mechanism of anti-tumour action between these antagonists. This is also supported by previous findings that co-administration of Cetrorelix and bombesin/GRP antagonists produces additive effects on inhibition of tumour growth (Yano et al 1993; Jungwirth et al 1997*a*; 1997*b*; 1998).

In an attempt to investigate the mechanism of action of bombesin/GRP antagonists, we tested the effect of GRP(14–27) on the expression of mRNA for c-*jun* and c-*fos* oncogenes, which are regulated by the bombesin-like peptides in small cell lung carcinomas and malignant glioblastomas (Draoui et al, 1995; Kiaris et

*© 2000 Cancer Research Campaign* 

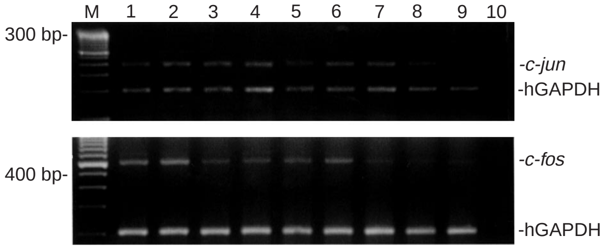

**Figure 6** Expression of c-*jun* and c-*fos* mRNA in OV-1063 tumours. Total RNA was extracted from tumours and c-*jun* and hGAPDH as well as c-*fos* and hGAPDH mRNAs were co-amplified by multiplex RT-PCR and electrophoresed on 2% agarose. Each RT-PCR reaction was repeated at least three times and similar results were obtained. Lanes 1–3 = expression of c-*jun* and c-*fos* mRNA in untreated tumours (control group); lanes 4–6 = expression of c-*jun* and c-*fos* mRNA in tumours from animals treated with RC-3940-II at a dose of 10 μg per day; lanes 7–9 = expression of c-*jun* and c-*fos* mRNA in tumours from animals treated with RC-3940-II at a dose of 20 μg per day; M = DNA molecular marker al, 1999). The exposure of OV-1063 cells cultured in vitro to GRP(14–27) resulted in a stimulation of mRNAs for c-*jun* and c-*fos* oncogenes. The stimulation was maximal at 1 h for c-*jun* and at 5 h for c-*fos*, while prolonged exposure to GRP(14–27) resulted in a lesser augmentation of the mRNA for these oncogenes. In OV-1063 cells cultured in vitro, the stimulation with GRP(14–27) caused a greater induction of mRNA expression for c-*jun* than for c-*fos*, in contrast to previous findings in SCLC (Draoui et al, 1995) and malignant glioblastomas (Kiaris et al, 1999). Considering that in all the three tumours GRPR/BRS-1 is the main receptor that mediates the proliferative effects of GRP(14–27), one might postulate that other factors acting downstream of each receptor modify the signal initiated by the ligand–receptor interaction, resulting in specific effects on c-*jun* or c-*fos* observed in each cell line. Bombesin/GRP antagonist RC-3940-II blocks the stimulatory action of GRP on c-*jun* and c-*fos* mRNAs, while the exposure of OV-1063 cells in vitro to RC-3940-II alone does not affect the mRNA levels of these oncogenes, indicating the absence of any intrinsic GRP activity in this antagonistic analogue of bombesin/GRP. This is in agreement with the finding that mRNA for GRP is not produced by OV-1063 cells and thus, the specific bombesin/GRP antagonist RC-3940-II, when added alone to the culture medium, has no effect in the regulation of c-*jun* and c-*fos* oncogenes, and the rate of cell proliferation. The detection of mRNA for the GRP ligand was performed using primers that span the common region of the prepro-GRP to the 3′-untranslated region of the message (Siegfried et al, 1994). Although the detection of the mRNA for GRP by more sensitive methods such as nested PCR and/or Southern blot hybridization cannot be excluded, the autocrine stimulation of this tumour by GRP might be much less important physiologically than paracrine stimulation.

In vivo, the levels of mRNA for c-*jun* and c-*fos* oncogenes decreased significantly in tumours of mice receiving RC-3940-II, which is likely due to the blockade of the endogenously produced mouse-derived GRP, since mRNA for GRP could not be detected in OV-1063 cells cultured in vitro. RC-3940-II administered at 20 μg per day but not at 10 μg per day inhibited significantly the levels of mRNA for c-*fos* and c-*jun* oncogenes in vivo. This is interesting because RC-3940-II, at both concentrations, produced significant inhibition of tumour growth. This finding probably indicates that higher levels of RC-3940-II are required for the suppression of c-*jun* and c-*fos* mRNA expression, than for inhibition of tumour growth.

The exact mechanism by which GRP stimulates the expression of c-*jun* and c-*fos* oncogenes is not completely understood. It has been shown that bombesin/GRP-like peptides stimulate phosphatidylinositol and $Ca^{2+}$ release (Spindel et al, 1993; Draoui et al, 1995), while the antagonistic analogues of bombesin/GRP downregulate the receptor for epidermal growth factor (Halmos et al, 1997) and inhibit the phosphorylation responses induced by bombesin-like peptides (Liebow et al, 1994). It is possible that the cascade of intracellular events initiated by the binding of bombesin/GRP-like peptides to specific membrane receptors results in the induction of c-*jun* and c-*fos* oncogenes. The products of c-*jun* and c-*fos* oncogenes form the AP-1 transcription factor, which in turn evokes the expression of genes with AP-1 inducible elements (Halazonetis et al, 1988). The overexpression of the AP-1 transcription factor is a common alteration detected in various cancers. Thus, the association of the anti-tumour activity of bombesin/GRP antagonists with a downregulation of c-*jun* and c-*fos* oncogenes and presumably the AP-1 levels would not be unexpected.

In summary, our results indicate that GRP is implicated in the pathogenesis of OV-1063 ovarian epithelial cell carcinoma and its mechanisms of action appears to involve the c-*jun* and c-*fos* oncogenes. Antagonistic analogues of bombesin/GRP could be considered for the treatment of ovarian epithelial cancers that depend on the production of bombesin-like peptides.

### AKNOWLEDGEMENTS

We thank Harold Valerio and Anita Feil for technical assistance and Dr H Kiaris for advice in the preparation of the manuscript. The work described in this paper was supported by the Medical Research Service of the Veterans Affairs department (to AVS) and by a grant from ASTA Medica (Frankfurt am Main, Germany) to Tulane University School of Medicine (to AVS).

### REFERENCES

Bajusz S, Csernus VJ, Janaky T, Bokser L, Fekete M and Schally AV (1988) New antagonists of LH-RH: II. Inhibition and potentiation of LH-RH by closely related analogues. *Int J Peptide Protein Res* **32**: 425–435

Bernhardt G, Reile H, Birnbock H, Sprub T and Schonenberger H (1992) Standardized kinetic microassay to quantify differential chemosensitivity on the basis of proliferative activity. *J Cancer Res Clin Oncol* **118**: 35–43

Bologna MC, Festuccia C, Muzi P, Biordi L and Ciomei M (1989) Bombesin stimulates growth of human prostatic cancer cells in vitro. *Cancer* **63**: 1714–1720

Cai RZ, Reile H, Armatis P and Schally AV (1994) Potent bombesin antagonists with C-terminal $Leu\psi(CH_2N)Tac\text{-}NH_2$ or its derivatives. *Proc Natl Acad Sci USA* **91**: 12664–12668

Carney DN, Cuttitta F, Moody TW and Minna JD (1987) Selective stimulation of small cell lung cancer clonal growth by bombesin and gastrin-releasing peptides. *Cancer Res* **47**: 821–825

Chegini N, Rong H, Dou Q, Kipersztok C and Williams ARS (1996) Gonadotropin-releasing hormone (GnRH) and GnRH receptor gene expression in human myometrium and leiomyomata and the direct action of GNRH analogs on myometrial smooth muscle cells and interaction with ovarian steroids in vitro. *J Clin Endocrinol Metab* **81**: 3215–3221

Cuttitta F, Carney DN, Mulshine J, Moody TW, Fedorko J, Fishler A and Minna JD (1985) Bombesin-like peptides can function as autocrine growth factors in human small-cell lung cancer. *Nature* **316**: 823–826

Draoui M, Chung P, Park M, Birrer M, Jakowlew S and Moody TW (1995) Bombesin stimulates c-*fos* and c-*jun* mRNAs in small cell lung cancer cells. *Peptides* **16**: 289–292

Emons G, Muller V, Ortmann O and Schulz K-D (1998) Effects of LH-RH-analogues on mitogenic signal transduction in cancer cells. *J Steroid Biochem Mol Biol* **65**: 199–206

 *© 2000 Cancer Research Campaign*

Halazonetis TD, Georgopoulos K, Greenberg ME and Leder P (1988) C-*jun* dimerizes with itself and with c-*fos*, forming complexes of different DNA binding affinities. *Cell* **55**: 917–924

Halmos G, Wittliff JL and Schally AV (1995) Characterization of bombesin/GRP receptors in human breast cancer and their relationship to steroid receptor expression. *Cancer Res* **53**: 280–287

Halmos G and Schally AV (1997) Reduction in receptors for bombesin and epidermal growth factor in xenografts of human small-cell lung cancer after treatment with bombesin antagonist RC-3095. *Proc Natl Acad Sci USA* **94**: 956–960

Horowitz AT, Treves AJ, Voss R, Okon E, Fuks Z, Davinson L and Biran S (1985) A new human ovarian carcinoma cell line: establishment and analysis of tumour associated markers. *Oncology* **42**: 332–337

Jungwirth A, Galvan G, Pinski J, Halmos G, Szepeshazi K, Cai RZ, Groot K and Schally AV (1997*a*) Luteinizing hormone-releasing hormone antagonist Cetrorelix (SB-75) and bombesin antagonist RC-3940-II inhibit the growth of androgen-independent PC-3 prostate cancer in nude mice. *Prostate* **32**: 164–172

Jungwirth A, Pinski J, Galvan G, Halmos G, Szepeshazi K, Cai RZ, Groot K, Vadillo-Buenfil M and Schally AV (1997*b*) Inhibition of growth of androgen-independent DU-145 prostate cancer in vivo by luteinising hormone-releasing hormone antagonist Cetrorelix and bombesin antagonists RC-3940-II and RC-3950-II. *Eur J Cancer* **33**: 1141–1148

Jungwirth A, Schally AV, Halmos G, Groot K, Szepeshazi K, Pinski J and Armatis P (1998) Inhibition of the growth of Caki-I human renal adenocarcinoma in vivo by luteinizing hormone-releasing hormone antagonist Cetrorelix, somatostatin analog RC-160, and bombesin antagonist RC-3940-II. *Cancer* **82**: 909–917

Kiaris H, Schally AV, Sun B, Armatis P and Groot K (1999) Inhibition of growth of human malignant glioblastoma in nude mice by antagonists of bombesin/gastrin-releasing peptide. *Oncogene* **18**: 7168–7173

Kim HJ, Evers MB, Banker NA, Greeley GH, Hellmich MR, Thompson JC and Townsend CM (1998) Novel expression and regulation of gastrin gene in human ovarian cancer cell line, SW626. *Dig Dis Sci* **43**: 1465–1473

Koppan M, Halmos G, Arencibia JM, Lambarzi N and Schally AV (1998) Bombesin/Gastrin-Releasing Peptide Antagonists RC-3095 and RC-3940-II inhibit tumour growth and decrease the levels and mRNA expression of epidermal growth factor receptors in H-69 small cell lung carcinoma. *Cancer* **83**: 1335–1343

Landis SH, Murray T, Bolden S and Wingo PA (1999) Cancer Statistics, 1999. *CA Cancer J Clin* **49**: 8–31

Levi F, Lucchini F, Negri E, Boyle P and La Vecchia C (1999) Cancer mortality in Europe, 1990–1994, and an overview of trends from 1955–1994. *Eur J Cancer* **35**: 1447–1516

Liebow C, Crean DH, Lee MT, Kamer AR, Mang TS and Schally AV (1994) Synergistic effects of bombesin and epidermal growth factor on cancers. *Proc Natl Acad Sci USA* **91**: 3804–3808

Markwalder R and Reubi JC (1999) Gastrin-releasing peptide receptors in the human prostate: Relation to neoplastic transformation. *Cancer Res* **59**: 1152–1159

Miyazaki M, Lamharzi N, Schally AV, Halmos G, Szepeshazi K, Groot K and Cai RZ (1998) Inhibition of growth of MDA-MB-231 human breast cancer xenografts in nude mice by bombesin/gastrin-releasing peptide (GRP) antagonists RC-3940-II and RC-3095. *Eur J Cancer* **34**: 710–717

Moody TW and Cuttitta F (1993) Growth factor and peptide receptors in small cell lung cancer. *Life Sci* **52**: 1161–1173

Nagalla SR, Barry BJ, Creswick KC, Eden P, Taylor JT and Spindel ER (1995) Cloning of a receptor for amphibian [Phe13]bombesin distinct from the receptor for gastrin-releasing peptide: Identification of a fourth bombesin receptor subtype (BR4). *Proc Natl Acad Sci USA* **92**: 6205–6209

Qin Y, Ertl T, Cai RZ, Halmos G and Schally AV (1994*a*) Inhibitory effect of bombesin receptor antagonist RC-3095 on the growth of human pancreatic cancer cells in vivo and in vitro. *Cancer Res* **54**: 1035–1041

Qin Y, Halmos G, Cai RZ, Szoke B, Ertl T and Schally AV (1994*b*) Bombesin antagonists inhibit in vitro and in vivo growth of human gastric cancer and binding of bombesin to its receptors. *J Cancer Res Clin Oncol* **120**: 519–528

Radulovic S, Cai RZ, Serfoso P, Groot K, Redding TW, Pinski J and Schally AV (1991) Biological effects and receptor binding affinities of new pseudononapeptide bombesin/GRP receptor antagonists with N-terminal D-Trp or D-Tpi. *Int J Pept Protein Res* **38**: 593–600

Reile H, Cai RZ, Armatis P and Schally AV (1995) New antagonists of bombesin/gastrin-releasing peptide with C-terminal Leu$\psi(CH_2N)$Tac-$NH_2$. *Int J Oncol* **7**: 749–754

Schally AV and Comaru-Schally AM (1997) Hypothalamic and other peptide hormones. In *Cancer Medicine* (4th edn), Holland JF, Frei III E, Bast Jr RC, Kufe DE, Morton DL and Weichselbaum RR (eds), pp 1067–1086. Williams and Wilkins: Baltimore

Shirahige Y, Cai RZ, Szepeshazi K, Halmos G, Pinski J, Groot K and Schally AV (1994) Inhibitory effect of bombesin/gastrin-releasing peptide (GRP) antagonists RC-3950-II and RC-3095 on MCF7-MIII human breast cancer xenografts in nude mice. *Biomed Pharmacother* **48**: 465–472

Siegfried JM, Han YH, DeMichele MAA, Hunt JD, Gaither AL and Cuttitta F (1994) Production of gastrin-releasin peptide by a non-small cell lung carcinoma cell line adapted to serum-free and growth factor-free conditions. *J Biol Chem* **269**: 8596–8603

Spindel ER, Giladi E, Segerson TP and Nagalla S (1993) Bombesin-like peptides: of ligands and receptors. *Recent Prog Horm Res* **48**: 365–391

Steel RGD and Torrie J (1976) *Principles and Procedures of Statistics*. McGraw Hill: New York

Sun B, Schally AV and Halmos G (2000) The presence of receptors for bombesin/GRP and mRNA for three receptor subtypes in human ovarian epithelial cancers. *Reg Peptides* **90**: 77–84

Sun B, Halmos G, Schally AV, Wang X and Martinez M (2000) The presence of receptors for bombesin/gastrin-releasing peptide and mRNA for 3 receptor subtypes in human prostate cancers. *Prostate* **42**: 295–303

Szepeshazi K, Schally AV, Halmos G, Groot K and Radulovic S (1992) Growth inhibition of estrogen-dependent and estrogen-independent MXT mammary cancers in mice by the bombesin and gastrin-releasing peptide antagonist RC-3095. *J Natl Cancer Inst* **84**: 1915–1922

Szepeshazi K, Schally AV, Halmos G, Lamharzi N, Groot K and Horvath JE (1997) A single in vivo administration of bombesin antagonist RC-3095 reduces the levels and mRNA expression of epidermal growth factor receptors in MXT mouse mammary cancers. *Proc Natl Acad Sci USA* **94**: 10913–10918

Tomayko MM and Reynolds CP (1989) Determination of subcutaneous tumour size in athymic (nude) mice. *Cancer Chemother Pharmacol* **24**: 148–154

UKCCCR (1998) United Kingdom Coordinating Committee on Cancer Research (UKCCCR) guidelines for the welfare of animals in experimental neoplasia (2nd edn). *Br J Cancer* **77**: 1–10

Wang QJ, Knezetic AJ, Schally AV, Pour PM and Adrian T (1996) Bombesin may stimulate proliferation of human pancreatic cancer cells through an autocrine pathway. *Int J Cancer* **68**: 1–7

Yano T, Pinski J, Groot K and Schally AV (1992) Stimulation by bombesin and inhibition by bombesin/gastrin releasing peptide antagonist RC-3950 of growth of human breast cancer cell lines. *Cancer Res* **54**: 4545–4547

Yano T, Pinski J, Szepeshazi K, Halmos G, Radulovic S, Groot K and Schally AV (1993) Inhibitory effect of bombesin/GRP antagonist RC-3095 and luteinizing hormone-releasing hormone antagonist SB-75 on the growth of MCF-7 MIII human breast cancer xenografts in athymic nude mice. *Cancer* **73**: 1229–1238

Yano T, Pinski J, Halmos G, Szepeshazi K, Groot K and Schally AV (1994) Inhibition of growth of OV-1063 human epithelial ovarian cancer xenografts in nude mice by treatment with luteinizing hormone-releasing hormone antagonist SB-75. *Proc Natl Acad Sci USA* **91**: 7090–7094

© 2000 Cancer Research Campaign
